# Essential involvement of the CX3CL1-CX3CR1 axis in bleomycin-induced pulmonary fibrosis via regulation of fibrocyte and M2 macrophage migration

**DOI:** 10.1038/s41598-017-17007-8

**Published:** 2017-12-04

**Authors:** Yuko Ishida, Akihiko Kimura, Mizuho Nosaka, Yumi Kuninaka, Hiroaki Hemmi, Izumi Sasaki, Tsuneyasu Kaisho, Naofumi Mukaida, Toshikazu Kondo

**Affiliations:** 1 Department of Forensic Medicine, Wakayama Medical University, Wakayama, Japan; 2 Department of Immunology, Institute of Advanced Medicine, Wakayama Medical University, Wakayama, Japan; 3 Division of Molecular Bioregulation, Cancer Research Institute, Kanazawa University, Kanazawa, Japan

## Abstract

The potential role of macrophages in pulmonary fibrosis (PF) prompted us to evaluate the roles of CX3CR1, a chemokine receptor abundantly expressed in macrophages during bleomycin (BLM)-induced PF. Intratracheal BLM injection induced infiltration of leukocytes such as macrophages into the lungs, which eventually resulted in fibrosis. CX3CR1 expression was mainly detected in the majority of macrophages and in a small portion of α-smooth muscle actin-positive cells in the lungs, while CX3CL1 was expressed in macrophages. BLM-induced fibrotic changes in the lungs were reduced without any changes in the number of leukocytes in *Cx3cr1* ^−/−^ mice, as compared with those in the wild-type (WT) mice. However, intrapulmonary CX3CR1^+^ macrophages displayed pro-fibrotic M2 phenotypes; lack of CX3CR1 skewed their phenotypes toward M1 in BLM-challenged lungs. Moreover, fibrocytes expressed CX3CR1, and were increased in BLM-challenged WT lungs. The number of intrapulmonary fibrocytes was decreased in *Cx3cr1* ^−/−^ mice. Thus, locally-produced CX3CL1 can promote PF development primarily by attracting CX3CR1-expressing M2 macrophages and fibrocytes into the lungs.

## Introduction

When tissue injuries occur due to microbial infection, toxic substances, or mechanical damages, inflammatory responses are initiated. Tissue repair occurs through the interplay between various hematopoietic and non-hematopoietic cells^1^. Deposition of extracellular matrix (ECM) components, such as collagen, is a crucial but reversible process in most cases of wound healing. However, if the tissue injury is severe or repetitive, or if the inflammatory response becomes dysregulated, progressive and irreversible accumulation of ECM may occur. The subsequent development of fibrosis can lead to organ failure, as seen in pulmonary fibrosis (PF), end-stage liver, kidney diseases, and heart failure^1^.

CX3CL1, also known as fractalkine, is a member of the CX3C chemokine family. It can bind to its specific receptor, CX3CR1, in a 1:1 ratio^2,3^. CX3CL1 is expressed by epithelial cells in the lungs, kidneys, and intestines as a membrane-bound molecule; its chemokine domain attaches to the cell surface through a mucin-like stalk in the resting state. CX3CR1 is abundantly expressed by monocytes/macrophages, T cells, natural killer cells, and smooth muscle cells^2,3^.

Accumulating evidence has demonstrated differential roles of CX3CL1-CX3CR1 interactions in various types of human diseases^4^. The CX3CL1-CX3CR1 system is suggested to promote the development of both atherosclerosis and coronary artery diseases^5,6^. It has also been reported that serum CX3CL1 levels correlate positively with disease severity in rheumatoid arthritis patients^7^. Furthermore, CX3CL1-CX3CR1 interactions could contribute to the pathogenesis of lung diseases, including asthma^8,9^ and emphysema^10,11^. On the contrary, CX3CL1-CX3CR1 signaling can either be beneficial or detrimental in carcinogenesis and cancer metastasis via alternative pathways^4^. Interestingly, studies have shown that the CX3CL1-CX3CR1 axis could protect against epilepsy, Parkinson’s disease, and amyotrophic lateral sclerosis^12^. Furthermore, the CX3CL1-CX3CR1 axis could maintain β cell function to preserve sufficient insulin secretion^13^. We have previously demonstrated that CX3CL1 protects against polymicrobial sepsis and toxin A-induced enteritis by binding to CX3CR1-expressing macrophages^14,15^.

PF is characterized pathologically by diffuse interstitial inflammation and fibrosis^16^. Moreover, PF can develop from various conditions, such as irradiation injury, oxygen toxicity-related pneumonitis, and scleroderma; however, approximately half of the PF cases are idiopathic. Environmental exposures to various substances^17^, as well as genetic factors^18^, have been proposed to be implicated in the development of idiopathic PF (IPF). However, their contribution to disease pathogenesis remains to be investigated. Aberrant tyrosine kinase activation in IPF has incited the clinical application of nintedanib, a multi-tyrosine kinase inhibitor. Moreover, pirfenidone, an agent with anti-inflammatory and anti-oxidant activities, was also examined for its effectiveness against IPF^19^. While both agents can delay the decline in respiratory function in IPF, they are unable to cure the disease. As a consequence, it is necessary to establish a novel treatment strategy based on the molecular and cellular mechanisms of IPF pathogenesis.

Bleomycin (BLM) can induce lung injuries that mimic the pathological features observed in human IPF. Analysis of BLM-induced lung injuries revealed the contribution of pulmonary macrophages to disease onset^20–24^. The expression of CX3CR1 on monocytes/macrophages prompted us to investigate the roles of the CX3CL1-CX3CR1 axis in BLM-induced lung injury in CX3CR1-deficient (*Cx3cr1* ^−/−^) mice. We revealed that genetic ablation of *Cx3xr1* significantly attenuated BLM-induced lung fibrosis.

## Results

### Intrapulmonary expression of CX3CL1 and CX3CR1 following BLM treatment

**Figure 1:**
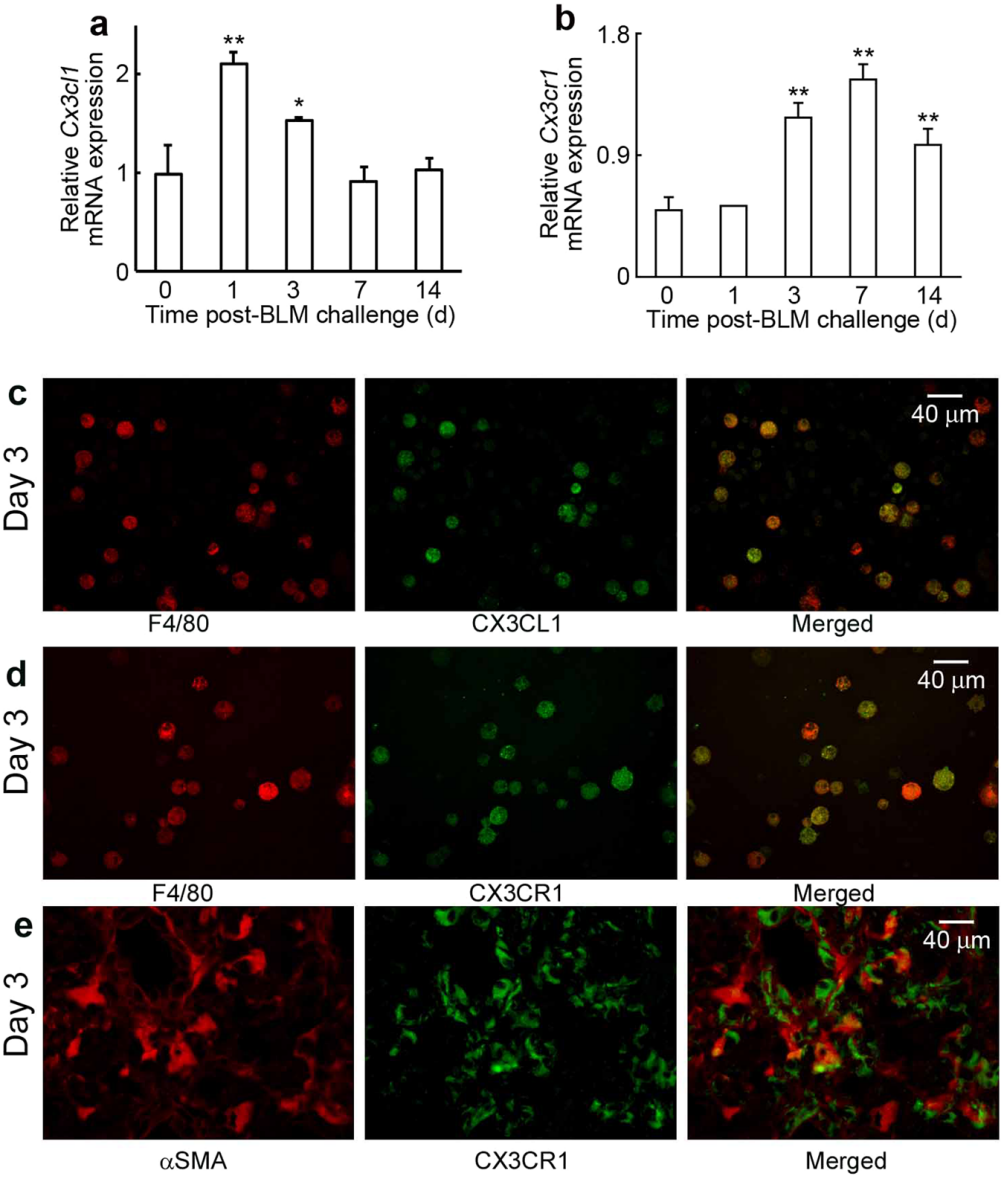
(**a**) and (**b**) Expression of *Cx3cl1* (**a**) and *Cx3cr1* (**b**) in the lung of WT mice after BLM challenge. Quantitative RT-PCR analyses of *Cx3cl1* and *Cx3cr1* gene expression was carried out. Values represent mean ± SEM (n = 6). *P < 0.05; **P < 0.01, vs. unchallenged lungs (time = 0). (**c**) to (**e**) Cell types expressing CX3CL1 and CX3CR1 in lungs of WT mice at 3 days after BLM exposure. Double-color immunofluorescence analyses were performed; representative images from six individual animals are shown here. Signals were merged digitally.

We first evaluated gene expression of *Cx3cl1* and *Cx3cr1* in lungs of WT mice following intratracheal BLM administration. Both *Cx3cl1* and *Cx3cr1* mRNAs were detected in lungs of untreated WT mice. Expression of *Cx3cl1* was significantly upregulated at 1 day and 3 days after the BLM challenge, followed by augmentation in *Cx3cr1* expression (Fig. 1a and b). Double-color immunofluorescence analysis demonstrated that F4/80^+^ cells, but not α-SMA^+^ fibroblasts and epithelial cells, were major cellular sources of CX3CL1 (Fig. 1c and Supp. Figure 1). Moreover, CX3CR1 proteins were detected in F4/80^+^ macrophages (Fig. 1d) and in a small proportion of α-SMA^+^ fibroblasts (Fig. 1e). Thus, intratracheal BLM injection induced F4/80^+^ cells mainly in the lungs to produce CX3CL1. Upregulation of CX3CL1 can attract F4/80^+^ macrophages and α-SMA^+^ fibroblasts through interactions with CX3CR1 expressed on these cells.

### Pathogenic roles of the CX3CL1-CX3CR1 axis in BLM-induced PF

**Figure 2:**
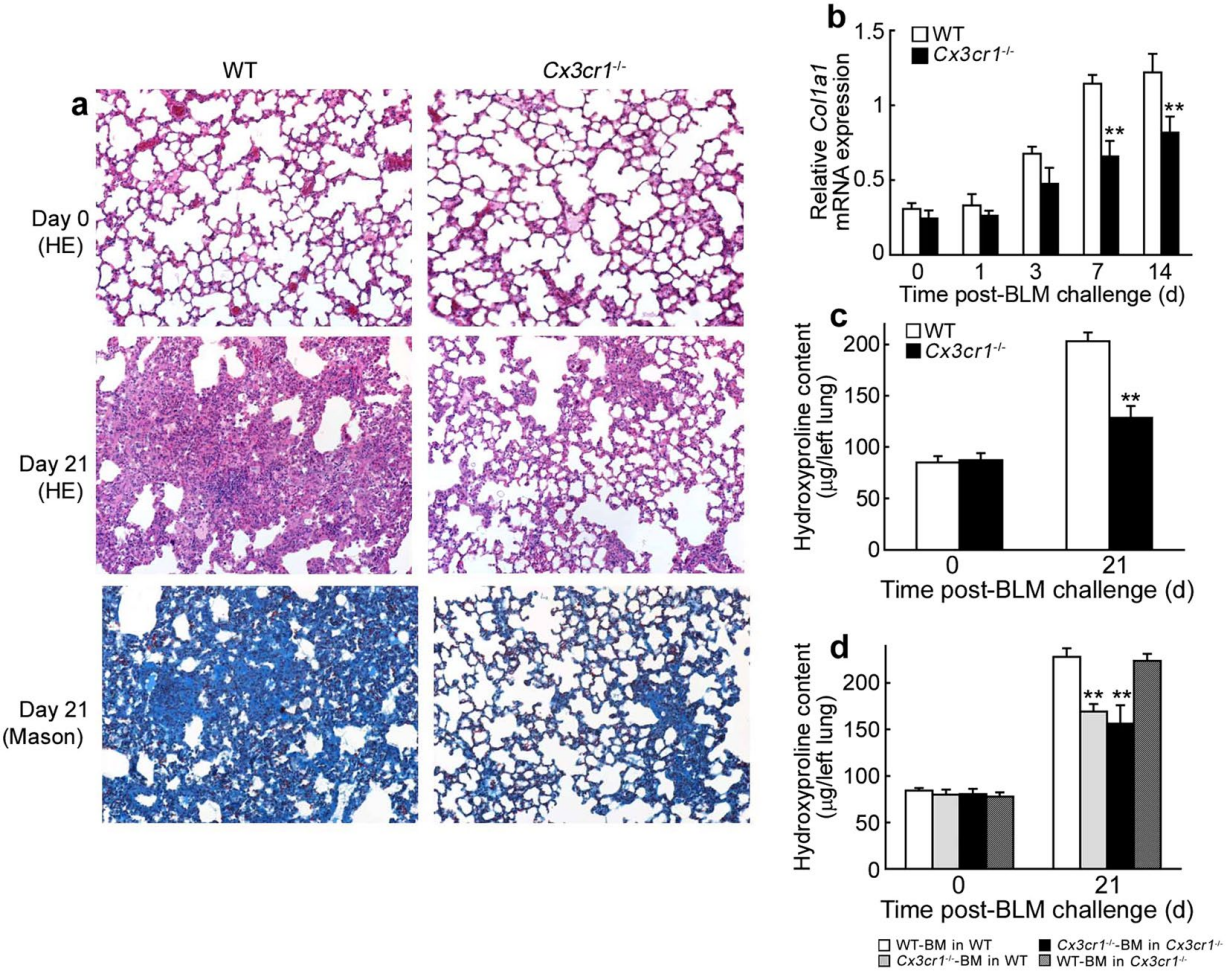
(**a**) Histopathological analysis of lungs from WT and *Cx3cr1* ^−/−^ mice after BLM treatment. Representative results from six animals at each time point are shown (HE stain and Masson’s stain). (**b**) Quantitative RT-PCR analyses of *Col1a1* in the lungs of WT and *Cx3cr1* ^−/−^ mice at the indicated time intervals after BLM treatment. All values represent mean ± SEM (n = 6). **P < 0.01, WT vs. *Cx3cr1* ^−/−^ mice. (**c**) Intrapulmonary Hyp content in WT and *Cx3cr1* ^−/−^ mice at 21 days after BLM challenge. All values represent mean ± SEM (n = 6). **P < 0.01, WT vs. *Cx3cr1* ^−/−^ mice. (**d**) BLM-induced PF in BM chimeric mice. Recipient female mice were transplanted with BM cells from *Cx3cr1* ^−/−^ or WT male donor. BM chimeric mice were injected with BLM at 60 days after BM transplantation. Hyp content in the lungs of BM chimeric mice was determined at 21 days after BLM exposure. All values represent mean ± SEM (n = 6). **P < 0.01, recipient with *Cx3cr1* ^−/−^BM cells vs. recipients with WT-BM cells.

In order to determine the pathophysiological roles of the CX3CL1-CX3CR1 axis in BLM-induced PF, we challenged WT and *Cx3cr1* ^−/−^ mice with intratracheal BLM. No significant histological differences were observed between the lungs of untreated WT and *Cx3cr1* ^−/−^ mice (Fig. 2a). At 21 days after BLM treatment, WT mice exhibited severe destruction of pulmonary alveolar structures and massive collagen deposition in the lungs, as evidenced by positive Masson staining (Fig. 2a). On the contrary, these fibrotic changes were markedly attenuated in *Cx3cr1* ^−/−^ mice (Fig. 2a). Moreover, *Col1a1* mRNA expression was significantly elevated in the lungs of WT mice at 7 and 14 days after BLM treatment; this enhancement was reduced in *Cx3cr1* ^−/−^ mice (Fig. 2b). The intrapulmonary content of hydroxyproline (Hyp), a major component of collagen, was also consistently increased in WT, but not *Cx3cr1* ^−/−^ mice, at 21 days after BLM treatment (Fig. 2c). We further challenged bone marrow (BM) chimeric mice generated from WT and *Cx3cr1* ^−/−^ mice with BLM, as CX3CR1 is expressed by BM- and non-BM-derived cells^25^. BLM treatment increased intrapulmonary Hyp content to a similar level as that in WT and *Cx3cr1* ^−/−^ mice with WT-derived BM (Fig. 2d). On the contrary, the increased Hyp content was reduced to a similar level in WT and *Cx3cr1* ^−/−^ mice with *Cx3cr1* ^−/–^-derived BM. Therefore, BM-derived CX3CR1^+^ cells may play a major role in the pathogenesis of BLM-induced PF.

### Involvement of the CX3CL1-CX3CR1 axis in intrapulmonary M2-macrophage infiltration

**Figure 3:**
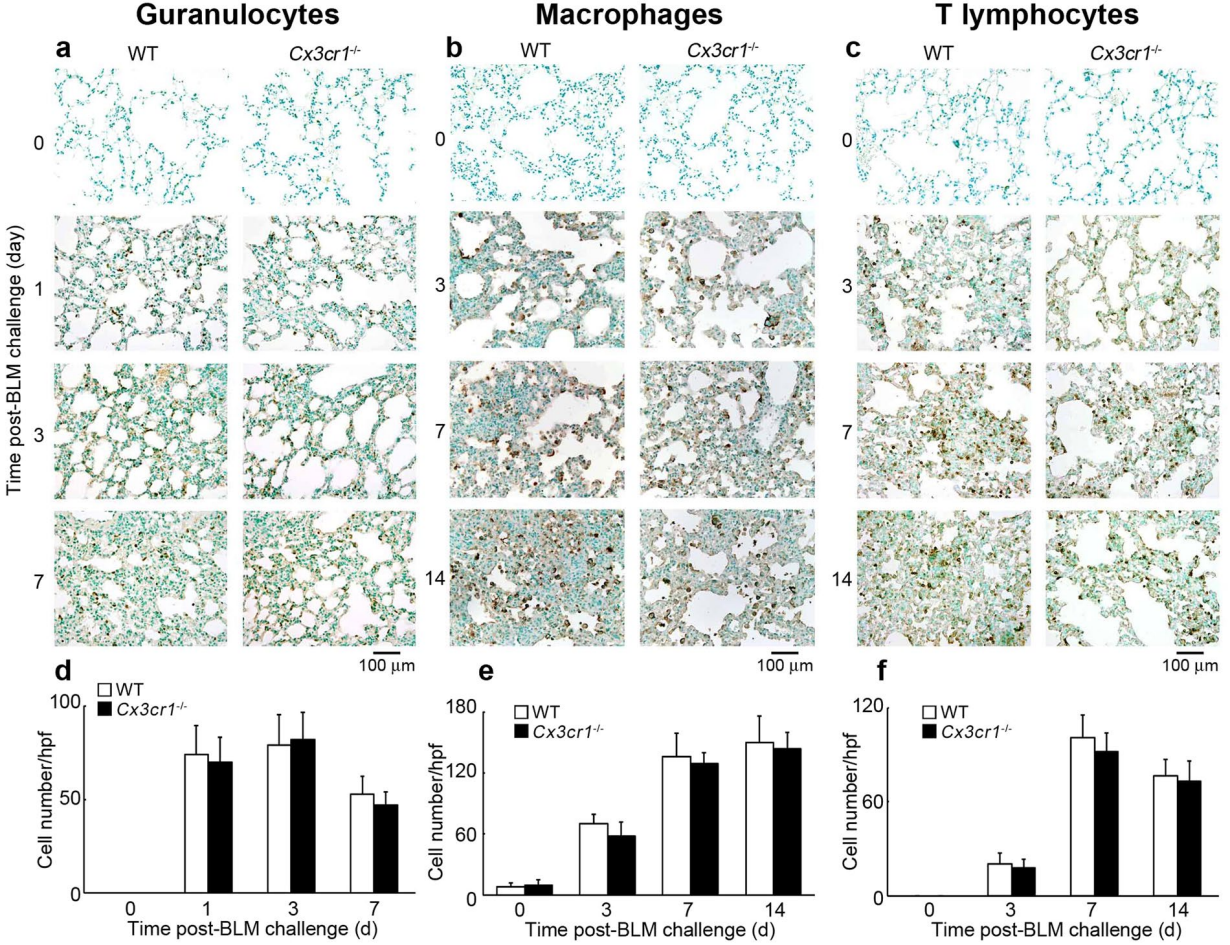
Immunohistochemical analyses of leukocyte numbers in WT and *Cx3cr1* ^−/−^ mouse lungs. (**a**) to (**c**) Immunohistochemical analyses were conducted using anti-Ly-6G (**a**) anti-F4/80 (**b**) or anti-CD3 Abs (**c**). Representative results from six individual animals are shown. (**d**) to (**f**) Number of granulocytes (**d**) macrophages (**e**) and T cells (**f**) in lung tissues was determined. All values represent mean ± SEM (n = 6).

**Figure 4:**
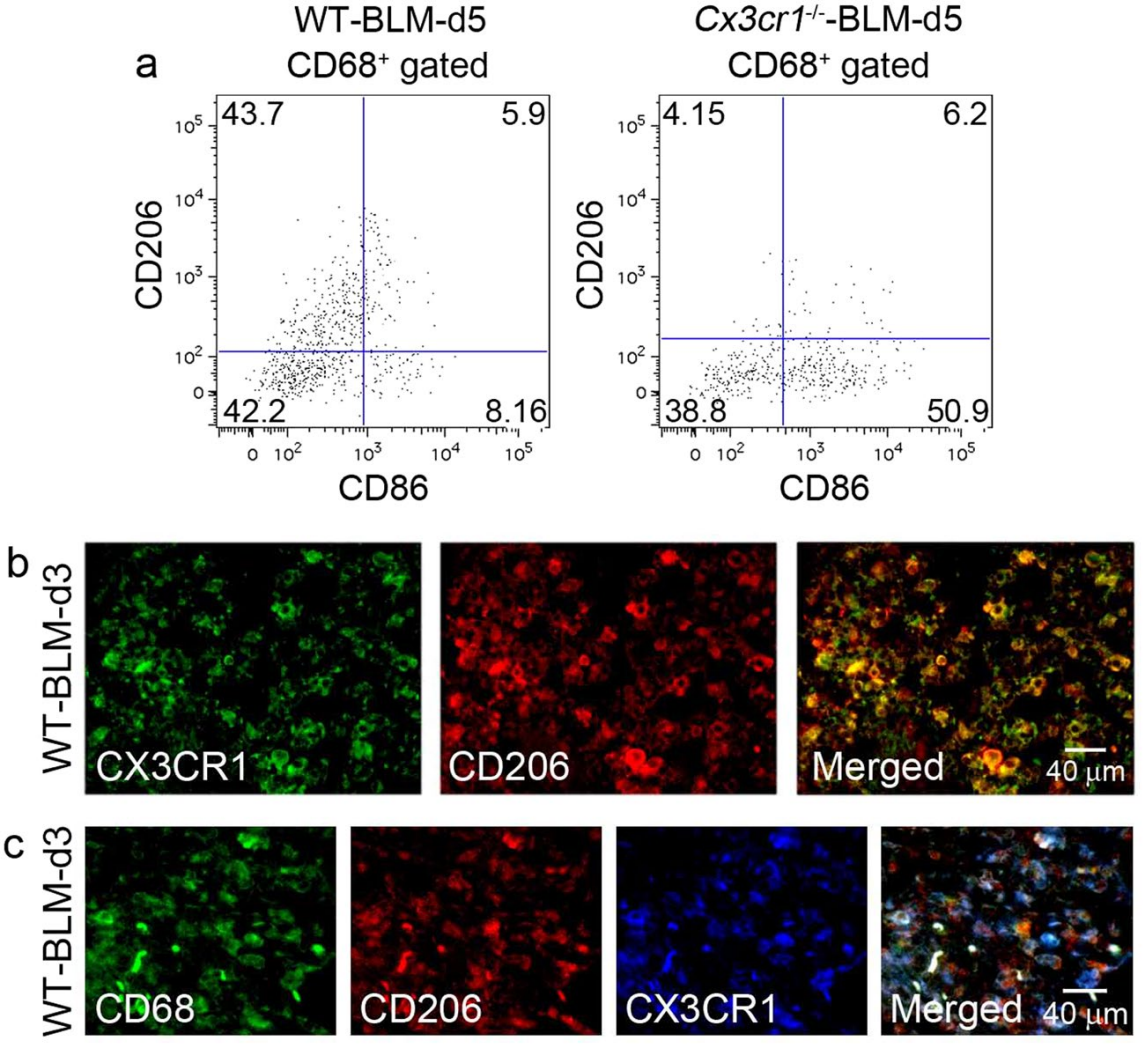
(**a**) Flow cytometric analysis of CD206^+^ M2-macrophages and CD86^+^ M1-macrophages among CD68^+^ macrophages in BALF from WT and *Cx3cr1* ^−/−^ mice at 5 days following BLM challenge. Representative results from three independent experiments with four animals in each group are shown. (**b**) Detection of CD206 on CX3CR1^+^ cells in the lungs of WT mice at 3 days after BLM exposure. (**c**) Detection of CX3CR1 on CD68^+^CD206^+^ M2-macropahges in the lungs of WT mice at 3 days after BLM exposure. Representative results from six individual animals are shown. Signals were merged digitally.

As leukocytes are hypothesized to play essential roles in the development of PF^26^, we wanted to evaluate leukocyte infiltration following BLM challenge. We did not observe any differences in the number of granulocytes, macrophages, and T cells between untreated WT and *Cx3cr1* ^−/−^ mice (Fig. 3). BLM challenge induced infiltration of granulocytes, macrophages, and T cells to similar extents in WT and *Cx3cr1* ^−/−^ mice (Fig. 3), which was confirmed at 5 days after BLM injection by flow cytometric analyses of bronchoalveolar lavage fluids (BALFs) (Supp. Figure 2). Therefore, absence of the CX3CL1-CX3CR1 axis may trigger compensatory enhancements in the expression of other chemokines, which lead to subsequent maintenance of inflammatory cell infiltration. However, this assumption was negated by the observation that absence of CX3CR1 had few impacts on the intrapulmonary expression of several chemokine and chemokine receptor genes, which were proposed to be involved in the pathogenesis of BLM-induced PF (Supp. Figure 3)^26^. Several lines of evidence demonstrated that functionally polarized macrophage subtypes, M1 and M2, are crucial for inflammation and tissue repair, respectively^27^. M2-macrophages are known to be important for tissue fibrosis^28^. As CX3CR1 was expressed by macrophages recruited into the lungs (Fig. 1d), we evaluated the phenotypes of BALF macrophages in WT and *Cx3cr1* ^−/−^ mice following BLM challenge. In the BALF of BLM-treated WT mice, the number of CD206^+^ macrophages (M2-type) dominated over CD86^+^ macrophages (M1-type). However, in BLM-treated *Cx3cr1* ^−/−^ mice, the opposite trend was observed (Fig. 4a). Immunofluorescence analysis demonstrated that CD206 and CD68 were simultaneously expressed by CX3CR1-expressing cells in the lung parenchyma after BLM challenge (Fig. 4b and c). Thus, the CX3CL1-CX3CR1 axis can play a crucial role in the intrapulmonary recruitment of pro-fibrotic M2-macrophages that eventually contribute to BLM-induced PF.

### Reduced intrapulmonary fibrocyte accumulation in *Cx3cr1*^−/−^ mice

**Figure 5:**
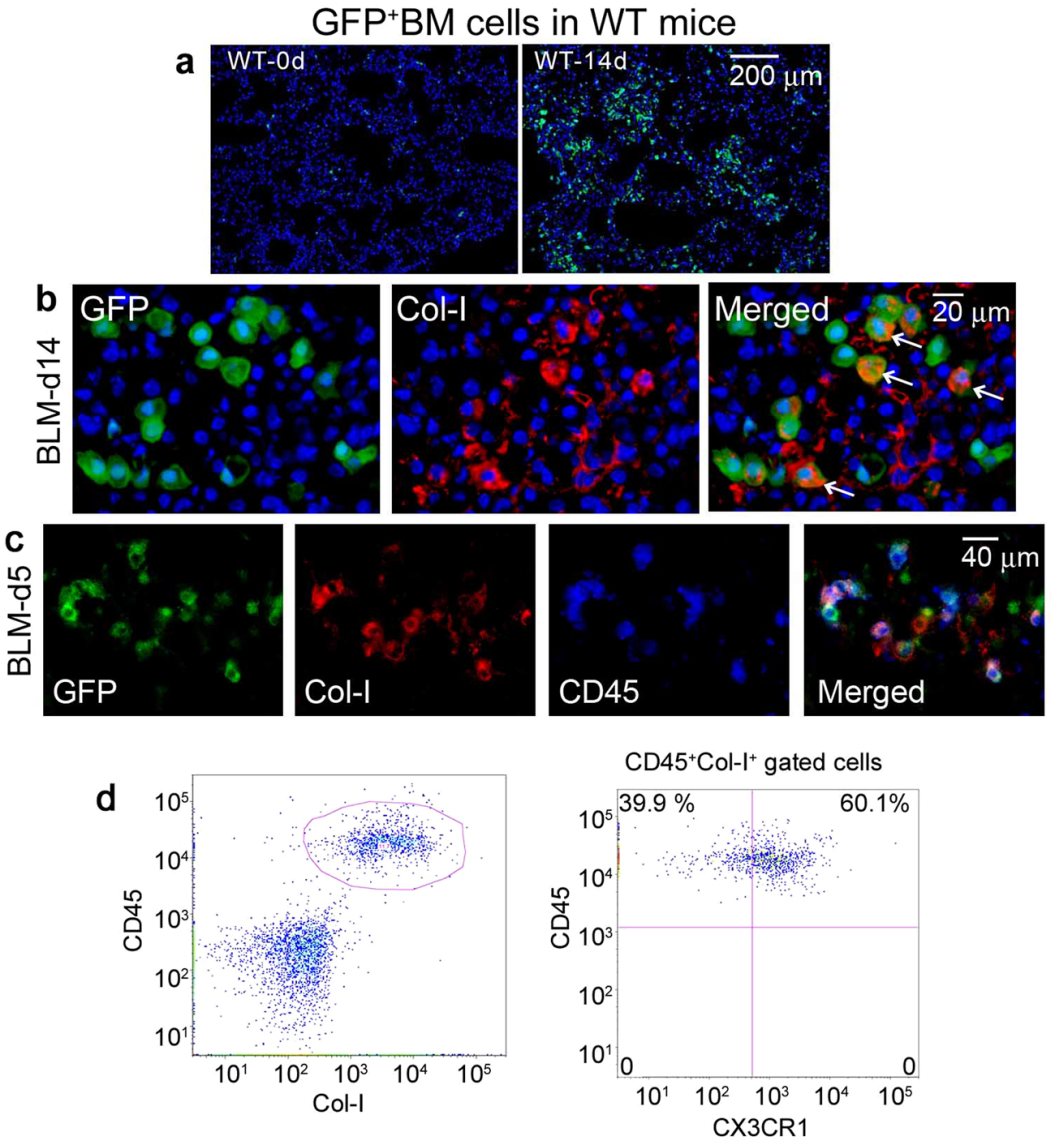
(**a**) Detection of BM-derived cells in the lungs after BLM treatment. BM chimeric mice were generated by transplanting BM cells of GFP-Tg mice to lethally irradiated WT mice. The resultant cells were challenged with BLM, and the lungs were obtained at 14 days after the challenge. Representative results from six individual animals are shown here. Nuclear staining is shown in blue. (**b**) Characterization of GFP^+^ BM cells recruited into the lungs after BLM challenge (at 14 days). Cells were immunostained with anti-Col-I and DAPI staining. (**c**) Characterization of GFP^+^ cells in the lung of WT mice at 5 days after BLM exposure. GFP^+^ cells were further treated with anti-Col-I and anti-CD45 antibodies. Representative results from 6 individual animals are shown. (**d**) Flow cytometric analysis of CX3CR1^+^ fibrocytes among CD45^+^Col-I^+^ fibrocytes in the BM of WT mice prior to BLM challenge. Representative results from 6 individual animals are shown here.

**Figure 6:**
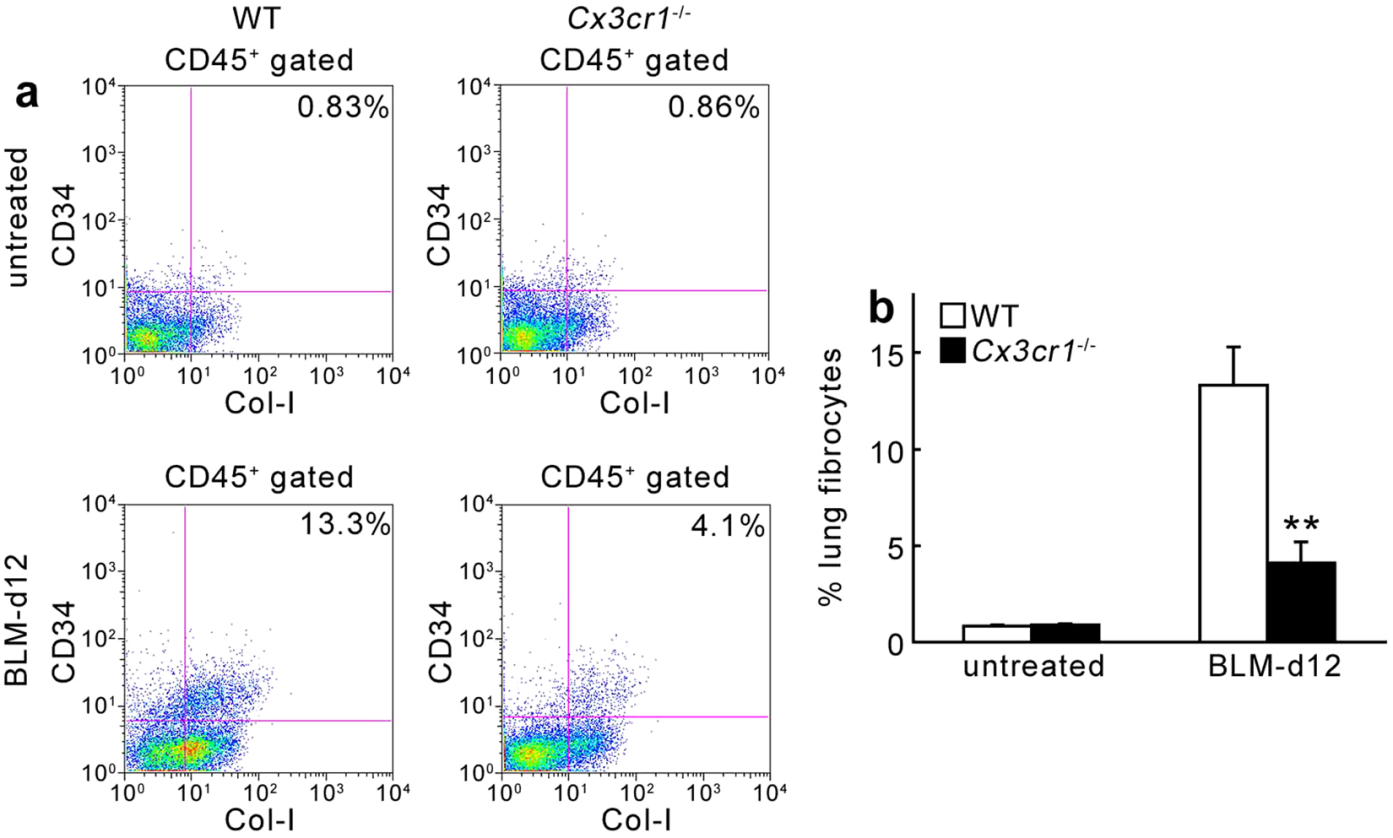
Quantitative evaluation of intrapulmonary fibrocytes by flow cytometric analyses at 12 days after BLM challenge. (**a**) Representative results from six independent experiments are shown here. (**b**) Changes in percentage of fibrocytes in lungs are shown. Values represent mean ± SEM (n = 6). **P < 0.01, WT vs. *Cx3cr1* ^−/−^.

Given the crucial involvement of BM-derived CX3CR1^+^ cells in lung fibrosis, we focused on an additional cell type present in the BM, the fibrocytes. These cells were identified as CD45^+^Col-I^+^ BM cells and hypothesized to be essential for tissue repair and abnormal fibrosis^29–31^. We generated BM chimeric mice by transplanting lethally irradiated WT mice with GFP-transgenic (Tg) mouse-derived BM cells. When WT mice bearing GFP^+^ BM cells were intratracheally injected with BLM, GFP^+^ cells was detected in the lungs, indicating that BM cells were recruited into the lungs after BLM treatment. In addition, Col-I was also detected in a substantial proportion of GFP^+^ cells (Fig. 5a and b). Moreover, immunofluorescence analyses revealed that both Col-I and CD45 were present in a large proportion of GFP^+^ cells recruited from the BM following BLM treatment (Fig. 5c). These observations suggested that BM-derived CD45^+^Col-I^+^ fibrocytes migrate to lungs after BLM treatment. Flow cytometry analysis revealed that CX3CR1 was expressed in approximately 60% of CD45^+^Col-I^+^ fibrocytes in the BM of WT mice after BLM treatment (Fig. 5d). We next determined the number of fibrocytes in the BM, peripheral blood, and lung tissues; there were no significant differences in fibrocyte numbers between untreated WT and *Cx3cr1* ^−/−^ mice (Fig. 6 and Supp. Figure 4). BLM challenge increased the number of fibrocytes to similar extents in the BM and peripheral blood of WT and *Cx3cr1* ^−/−^ mice (Supp. Figure 4). Number of fibrocytes in the lungs were markedly increased in WT mice at 12 days after BLM treatment. However, this enhancement in the number of fibrocytes was suppressed in *Cx3cr1* ^−/−^ mice (Fig. 6). Collectively, these results suggested that locally-produced CX3CL1 can contribute to BLM-induced PF by recruiting CX3CR1-expressing fibrocytes that produce Col-I from the BM into the lungs.

### Reduced TGF-β1 expression in mice lacking CX3CR1

**Figure 7:**
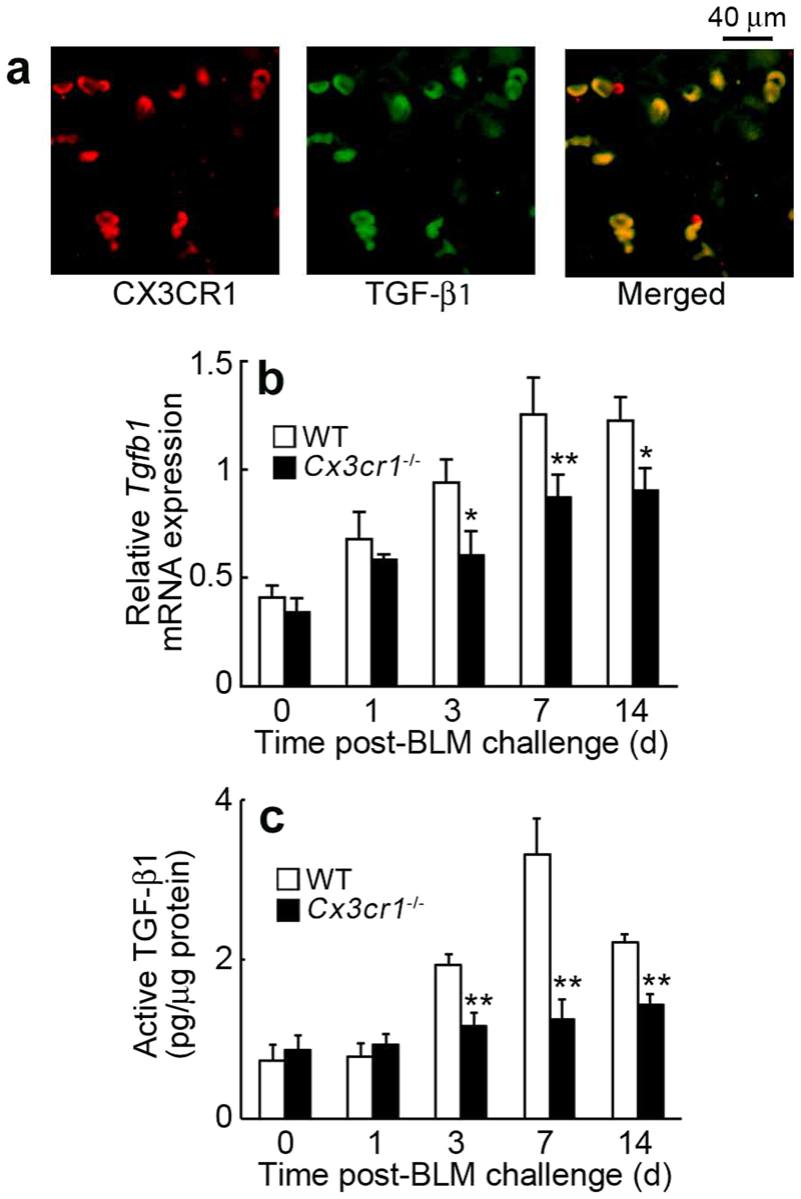
Intrapulmonary TGF-β1 expression in the lungs after BLM treatment. (**a**) Cell types expressing TGF-β1 in the lungs of BLM-treated WT mice. Double-color immunofluorescence analyses were performed. Representative results from six individual animals are shown here. Signals were merged digitally. (**b**) Intrapulmonary TGF-β1 expression in WT and *Cx3cr1* ^−/−^ after BLM challenge. *Tgfb1* mRNA expression was analyzed by quantitative RT-PCR. (**c**) Active TGF-β1 protein content in the lungs was determined with ELISA. Values represent mean ± SEM (n = 6). *P < 0.05; **P < 0.01, WT vs. *Cx3cr1* ^−/−^ mice.

TGF-β1 is a potent fibrogenic growth factor in various organs. We examined the cell types expressing TGF-β1 in the lungs after BLM treatment. Immunofluorescence analyses showed expression of TGF-β1 in CX3CR1^+^ cells (Fig. 7a), which suggested that CX3CR1-expressing cells, fibrocytes, and M2-macrophages could be a major source of TGF-β1 in the lungs. Moreover, BLM treatment progressively enhanced TGF-β1 expression at both the gene and protein levels in the lungs of WT mice; this enhancement was markedly suppressed in the lungs of *Cx3cr1* ^−/−^ mice (Fig. 7b and c). As a result, the CX3CL1-CX3CR1 axis can have an indispensable role in BLM-induced lung fibrosis by inducing the migration of TGF-β1-producing fibrocytes and M2-macrophages into the lungs.

## Discussion

It is highly evident from previous studies that macrophages are indispensable in tissue repair and the subsequent fibrotic changes^32,33^. Robust CX3CR1 expression in macrophages^2,3^ prompted several scientific groups to investigate the roles of the CX3CL1-CX3CR1 axis in fibrosis in various organs. Studies have shown that hypertension- and unilateral ureteral obstruction-induced renal fibrosis are attenuated by *Cx3cr1* gene deficiency^34–36^. On the contrary, the interplay between CX3CL1 and CX3CR1 can prevent carbon tetrachloride-induced liver injury^37,38^. These discrepancies may be explained by organ-specific differences in macrophage populations; CX3CR1-expressing macrophages may exert anti-inflammatory activities, as observed in corneal tissues^39^. Hence, we examined the effect of CX3CR1 deficiency on BLM-induced PF. Our results revealed that CX3CR1 deficiency inhibits BLM-induced lung pathologies, but does not exert any effect on infiltration of inflammatory cells, including macrophages.

Macrophages in the lungs belong to two subpopulations, interstitial macrophages (IMs) and alveolar macrophages (AMs), residing in the interstitial and alveolar spaces, respectively^40^. Ly6^high^CCR2^+^ inflammatory monocytes in the circulation are recruited into lung tissues, most likely in a CCL2-dependent manner, similar to lung metastasis in breast cancer^41^. On the contrary, AMs originate from fetal liver monocytes, and self-maintain throughout their life cycle under steady state^42^. Allergen challenges reduced the number of embryo-derived AMs^43^ and enhanced CCL2 expression-induced migration of CCR2-expressing monocyte-derived AMs to replenish the AM loss^44^. Therefore, total number of intrapulmonary macrophages may not differ significantly between BLM-treated WT and *Cx3cr1* ^−/−^ mice, since BLM may be able to enhance the intrapulmonary expression of the chemoattractant CCL2 for both IMs and AMs to similar extents.

Alternatively, there remains a possibility that CX3CR1 deficiency may have profound impact on macrophage functions. Macrophages can be functionally polarized into two phenotypes, M1 and M2. Skewing of macrophage polarization can contribute to pathogenesis of inflammatory responses, allergic diseases, tissue repair, and tumor progression^45–47^. Several lines of evidence indicated that macrophages with the M2 phenotype could promote tissue fibrosis^48,49^. Indeed, CX3CR1-expressing CD68^+^ cells in the lungs were also positive for CD206^50,51^, a M2-specific marker. This indicated that CX3CR1^+^ macrophages are M2-macrophages. Moreover, M2-macrophages were reduced in BLM-challenged lungs of *Cx3cr1* ^−/−^ mice. This was concomitant with a reciprocal increase in CD68^+^ M1-macrophages. Therefore, absence of CX3CR1 can have profound effects on macrophage polarization rather than total macrophage numbers in the lungs after BLM challenge.

Myofibroblasts display characteristics of both fibroblasts and smooth muscle cells, as represented by their ECM-synthesizing capacity and contractile cytoskeleton, respectively^52^. As a result, myofibroblasts have indispensable roles in connective tissue remodeling and the subsequent development of fibrosis in various organs, including the lung. Myofibroblasts can be differentiated from several cell types, including locally residing mesenchymal cells (e.g., fibroblasts and smooth muscle cells), other local sources such as epithelial and endothelial cells, and BM-derived fibrocytes^53^. Studies on BM chimeric mice have revealed the crucial role of radiosensitive BM-derived CX3CR1-expressing cells in BLM-induced collagen deposition in the lung. Moreover, injection of BLM into chimeric mice with BM derived from GFP Tg mice resulted in cellular expression of GFP^+^ and Col-I^+^ in the lung. Therefore, fibrocytes may be a major source of collagen-producing myofibroblasts in BLM-induced PF.

Fibrocytes are defined as BM-derived cells that are intermediate between hematopoietic and mesenchymal cells, as they simultaneously express leukocyte markers such as CD45 and mesenchymal cell markers such as Col-I. Fibrocytes, once generated in the BM, circulate in the bloodstream, and migrate to organs in response to tissue-specific cues^29–31,54–62^. BLM was found to increase fibrocyte numbers in the BM, peripheral blood, and lungs, which is consistent with our previous report^26^. Moreover, fibrocytes were hypothesized to express several chemokine receptors, including CXCR4, CCR3, CCR5, and CCR7^31,63,64^, which allow them to migrate to fibrotic organs in response to their cognate ligands. It was found that inhibition of CXCR4 function^31^, or deficiencies in *Ccr3* ^65^ or *Ccr5* ^26^ attenuated BLM-induced pathologies, together with reduced intrapulmonary fibrocyte numbers. Our present observations suggested that CX3CR1 is one of the chemokine receptors involved in migration of fibrocytes into fibrotic lungs.

We have previously demonstrated that *Ccl3* ^−/−^ and *Ccr5* ^−/−^ mice exhibit reduction in BLM-induced fibrosis and the number of CD45^+^Col-I^+^CCR5^+^ fibrocytes in the lungs, together with suppressed CXCL12 expression^26^. These observations indicated that the CCL3-CCR5 axis can mediate BLM-induced fibrocyte migration into the lungs, partly via its interactions with the CXCL12-CXCR4 axis. BLM-induced increase in intrapulmonary fibrocyte numbers was not completely nullified in *Cx3cr1* ^−/−^ mice, suggesting the contribution of other chemokines such as CXCL12 and CCL3 to fibrocyte infiltration. The decreased BLM-induced fibrosis and fibrocyte infiltration into the lungs of *Cx3cr1* ^−/−^ mice, however, were not accompanied with changes in intrapulmonary expression of CXCL12 and ligands for CCR5, CCL3, CCL4, and CCL5. Thus, the CX3CL1-CX3CR1 axis can regulate BLM-induced fibrocyte migration and subsequent fibrosis development independently of other chemokines.

CX3CL1 has distinct structural and functional features from other chemokines. Most chemokines are produced as a secretory molecule, and bind to proteoglycan and glycosaminoglycan in the ECM through their carboxy-terminal α-helix portion^66^; this binding is reversible and unstable. On the contrary, CX3CL1 is expressed as a membrane-bound molecule with a mucin-like stalk^2,3^. As compared with other chemokines, CX3CL1 can be firmly retained on the cell surface, and can consequently affect its target cells in a more stable manner. Moreover, in addition to chemotactic activities, CX3CL1-CX3CR1 interactions can promote the survival and/or proliferation of non-hematopoietic cells, such as neuronal cells^67^, and hematopoietic cells, such as monocytes^68^ and helper T cells^9^. If CX3CL1 is able to support survival and/or proliferation of fibrocytes as well as non-hematopoietic cells, it can attract CX3CR1-expressing fibrocytes into the lung. This can eventually promote the survival and/or proliferation of fibrocytes, thereby inducing fibrotic changes.

Collectively, our observations suggested that the CX3CL1-CX3CR1 axis is essential in the development of BLM-induced PF by regulating fibrocytes and M2-macrophags, which can exert pro-fibrotic activities. Absence of CX3CR1 impairs wound healing in the skin with reduced macrophage recruitment and fibroblast accumulation^10^. To summarize, the interactions between CX3CL1 and CX3CR1 may provide potential candidate molecule(s) for the treatment of IPF.

## Methods

### Reagents and antibodies (Abs)

BLM was purchased from Sigma Chemical Co. (St. Louis, MO). The following monoclonal antibodies (mAbs) and polyclonal antibodies (pAbs) were used for immunohistochemical and immunofluorescence analyses in this study: goat anti-mouse CX3CL1 pAbs and goat anti-mouse CX3CR1 pAb (Santa Cruz Biotechnology, Santa Cruz, CA); rabbit anti-human CX3CR1 pAbs, which cross-reacts with mouse CX3CR1 (Abnova, Walnut, CA); rat anti-mouse F4/80 mAb (clone, BM8; BMA Biomedicals, Switzerland); mouse anti-human α-smooth muscle actin (α-SMA) mAb (clone, 1A-4), rabbit anti-mouse pan-Cytokeratin pAbs (Santa Cruz Biotechnology), rabbit anti-CD68 (BIO RAD, Hercules, CA), rat anti-CD206 (Abcam, Cambridge, UK), rabbit anti-human CD3 pAbs, which cross-reacts with mouse CD3 (Dako Cytomation, Kyoto, Japan); rat anti-mouse Ly-6G mAb, (clone, 1A8; BD Biosciences, San Jose, CA); rat anti-mouse F4/80 mAb (clone, A3-1; AbD Serotec, Oxford, UK); rabbit anti-mouse collagen type I (Col-I) pAbs (Merck Millipore, Billerica, MA); rat-anti mouse TGF-β1 mAb (clone, 860206; R&D, Minneapolis, MN), Cy3-conjugated donkey anti-rat, -goat, -mouse, and -rabbit IgG pAbs, and FITC-conjugated donkey anti-goat, -rat, and -rabbit IgG pAbs (Jackson ImmunoResearch Laboratories, West Grove, PA). For flow cytometric analysis, the following antibodies (Abs) were commercially obtained: PerCP-conjugated rat anti-mouse CD45 mAb (clone, 30-F11) and FITC-conjugated rat anti-mouse CD34 mAb (clone, RAM34; BD Biosciences); rabbit anti-mouse Col-I pAbs (Merck Millipore, Billerica, MA); PE-conjugated rabbit anti-mouse CX3CR1 (PromoKine, Heidelberg, Germany); FITC-conjugated rat anti-mouse CD68 (BIO RAD, Hercules, CA); PE-conjugated rat anti-mouse CD86, FITC-conjugated rat anti-mouse CD8, FITC-conjugated Armenian hamster anti-mouse CD3, PE-conjugated mouse anti-mouse NK1.1, FITC-conjugated rat anti-mouse CD11b (BD Biosciences, Franklin Lakes, NJ); APC-conjugated rat anti-mouse CD206 (R&D, Minneapolis, MN); and FITC-conjugated rat anti-mouse Gr-1, PE-conjugated rat anti-mouse CD4 (Thermo Fisher Scientific, Waltham, MA).

### Mice

Pathogen-free 8-week-old male C57BL/6 mice were obtained from Sankyo Laboratories (Tokyo, Japan), and were designated as wild-type (WT) mice. *Cx3cr1* ^−/−^ mice on the C57BL/6 genetic background were a generous gift from Drs. P.M. Murphy and J.L. Gao (National Institute of Allergy and Infectious Diseases, National Institutes of Health, Bethesda, MD)^25^. Tg mice on the C57BL/6 genetic background were obtained from RIKEN BRC (Tsukuba, Japan). All animals were housed individually in cages under specific pathogen-free conditions during the experiments. Age- and sex-matched mice were used for the experiments. All animal experiments complied with the standards set out in the guidelines for the Care and Use of Laboratory Animals at the Wakayama Medical University.

### BLM-induced lung Injury

Mice were put under deep anesthesia with an intraperitoneal injection of pentobarbital (50 µg/g weight). A cervical midline incision was made, and the trachea was exposed. Thereafter, BLM (0.075 U) in 50 µl sterile saline was intratracheally administered with a 26-guage needle. At the indicated time intervals following BLM administration, mice were sacrificed by pentobarbital overdose, and both lungs were removed for subsequent analyses.

### Generation of bone BM chimeric mice

BM chimeric mice were prepared as previously described^26^. Briefly, male *Cx3cr1* ^−/−^ BM → female WT mice, male WT BM → female *Cx3cr1* ^−/−^ mice, male WT BM → female WT mice, and male *Cx3cr1* ^−/−^ BM → female *Cx3cr1* ^−/−^ mice. BM cells were collected from femurs of donor mice by aspiration and flushing. Recipient mice were irradiated to 15 Gy with a RX-650 irradiator (Faxitron X-ray Inc., Wheeling, IL). The animals then received intravenous injections of 5 × 10^6^ BM cells in 200 μl sterile PBS(−) from donor mice under anesthesia. Thereafter, the mice were housed in sterilized microisolator cages, and were fed normal chow and autoclaved hyperchlorinated water for 60 days. Similarly, BM chimeric mice were generated by transplanting GFP-Tg mouse-derived BM cells into lethally irradiated WT mice. To verify successful engraftment and reconstitution of the BM in transplanted mice, genomic DNA was isolated from peripheral blood and tail tissues of each chimeric mouse at 30 days after BMT with a NucleoSpin tissue kit (Macherey-Nagel, Duren, Germany). Polymerase chain reaction (PCR) was used to detect the *Sry* gene in the Y chromosome (forward primer, 5′-TTGCCTCAACAAAA-3′; reverse primer, 5′-AAACTGCTGCTTCTGCTGGT-3′). The amplified PCR products were fractionated on a 2% agarose gel, and were visualized by ethidium bromide staining. After durable BM engraftment was confirmed, mice were treated with BLM, as described above.

### Histopathological and immunohistochemical analyses

Lung tissues were fixed in 10% formalin buffered with PBS (pH 7.2), and were embedded in paraffin. Tissues were sectioned (6 µm-thick) and stained with hematoxylin and eosin or Masson’s trichrome to detect collagen deposition. Immunohistochemical analyses were also performed using anti-Ly6G, anti-F4/80, or anti-CD3 Abs, as previously described^26^. Cells were quantified in 10 randomly chosen visual fields at 200× magnification, and the average of the 10 selected microscopic fields was calculated. All measurements were performed by an examiner without prior knowledge of the experimental procedures.

### Double- and triple-color immunofluorescence analysis

Double-color immunofluorescence analysis was conducted to identify the types of CX3CL1- or CX3CR1-expressing cells in the lung, as previously described^69^. Briefly, deparaffinized sections were incubated with PBS containing 1% normal donkey serum and 1% BSA to reduce nonspecific reactions. Tissue sections were then incubated with a combination of anti-CX3CR1 and anti-F4/80 Abs, anti-CX3CL1 and anti-F4/80 Abs, or anti-CX3CR1 and anti-α-SMA Abs at 1 µg/ml at 4 °C overnight. Similarly, triple-color immunofluorescence analysis was conducted to identify the molecules expressed on BM-derived fibrocytes (GFP^+^Col-I^+^ cells), such as TGF-β1 and CX3CR1. Sections containing GFP^+^ cells were incubated with a combination of anti-Col-I and anti-CD45 Abs, anti-Col-I and anti-TGF-β1 Abs, or anti-Col-I and anti-CX3CR1 Abs at a concentration of 1 µg/ml at 4 °C overnight. In order to identify the subtypes of macrophages recruited into the lungs, tissue sections were incubated with a combination of anti-CD68, anti-CX3CR1, and anti-CD206 Abs, or anti-CD206 and anti-CX3CR1 Abs at a concentration of 1 mg/ml at 4 °C overnight. Following incubation with fluorochrome-conjugated secondary Abs, tissues were imaged under a fluorescence microscope.

### Determination of Hyp content

Lung tissues were removed at 21 days after BLM administration to determine the content of Hyp, a major component of collagen, as previously described^26^. Data were expressed as the amount (μg) of Hyp per lung.

### Flow cytometric analysis of leukocytes in BALF

BALF was collected as previously described with some modifications^26^. Briefly, mice were sacrificed by abdominal aorta dissection. The trachea was cannulated, and the airway lumen was washed two times with 1 ml ice-cold PBS. Single cell suspensions were incubated with 25 µg/ml Fc block (BD Biosciences, Piscataway NJ) for 15 min on ice to prevent nonspecific binding. Cells were stained with anti-CD68, anti-CD86, CD-206, anti-CD3, anti-CD4, anti-CD8, anti-NK1.1, anti-Gr-1, and anti-CD11b. Analysis was performed on a FACScan flow cytometer (BD Biosciences) using the FlowJo software (Tommy Digital Biology, Tokyo, Japan).

### Quantitative RT-PCR analysis

Total RNA was extracted from lung tissue using ISOGEN (Nippon Gene, Toyama, Japan), according to the manufacturer’s instructions. Total RNA (3 µg) was reverse transcribed to cDNA using PrimeScript™ Reverse Transcriptase (Takara Bio, Shiga, Japan) with Oligo(dT)_15_ primers. Quantitative PCR was carried out with the resultant cDNA as templates and SYBR^®^ Premix Ex Taq™ II (Takara Bio) with specific primer sets, as previously described^69^ (Supplemental Table 1). Primers were purchased from Takara Bio. Amplification and detection of mRNA were conducted using the Thermal Cycler Dice^®^ Real Time System (Takara Bio, TP800), according to the manufacturer’s instructions. Expression levels of target genes were analyzed using the ΔΔCt comparative threshold method. The *β-actin* gene was used as internal control.

### Determination of fibrocytes

Mononuclear cells were isolated from the lungs, the BM, and peripheral blood, as previously described^25^. Nonspecific binding was blocked by incubation with 25 µg/ml Fc block (BD Biosciences Pharmingen, Piscataway, NJ) for 15 min at 4 °C. Cells were stained with PerCP-labeled anti-CD45 and FITC-labeled anti-CD34. After extensive washing with PBS(−), cells were permeabilized using cytofix/cytoperm (BD Biosciences Pharmingen), stained with anti-Col-I pAbs, and incubated with PE-conjugated goat anti-rabbit Ig (Life Technologies, Grand Island, NY), as previously described^26^. After two PBS(−) washes, cells were fixed in 2% paraformaldehyde. Isotype-matched control immunoglobulins were used to detect non-specific binding. Stained cells were analyzed on the FACS Calibur flow cytometer. In parallel, BM cells from BLM-treated WT mice were stained with anti-Col-I, anti-CD45, and anti-CX3CR1 to determine the proportion of CX3CR1^+^ fibrocytes. Flow cytometry was performed on a FACS Calibur flow cytometer, and data were analyzed using the FlowJo software.

### ELISA

Lung samples from WT mice were homogenized with PBS containing complete protease inhibitor cocktail (Roch Diagnostics, Mannheim, Germany). Homogenates were centrifuged at 10,000 × *g* for 10 min. Supernatants were used to quantify the active form of TGF-β1 with a commercial ELISA kit (R&D Systems), according to manufacturer’s instructions. The detection limits was 7 pg/ml. Total protein in the supernatant was measured with a commercial kit (BCA protein assay kit, Pierce, Rockford, IL). Data were expressed as TGF-β1 (pg) per total protein (µg) for each sample.

### Statistical analysis

The means and SEMs were calculated for all investigated parameters in the study. Statistical significance was evaluated by ANOVA or the Mann-Whitney’s *U*-test. *P* < 0.05 was accepted as being statistically significant.

### Study approval

All animal experiments were approved by the Committee on Animal Care and Use at the Wakayama Medical University. All methods were performed in accordance with relevant guidelines and regulations.

### Data availability

Data that support the findings of this study are available from the corresponding author upon reasonable request.

## Electronic supplementary material

Supplementary Information
