# Supplementary material for: Essential involvement of the CX3CL1-CX3CR1 axis in bleomycin-induced pulmonary fibrosis via regulation of fibrocyte and M2 macrophage migration
Source: Sci Rep. 2017 Dec 4;7:16833. doi: 10.1038/s41598-017-17007-8 (PMC5714949; doi:10.1038/s41598-017-17007-8)
Supplement: Supplementary file 1 — Supplementary Information [file 41598_2017_17007_MOESM1_ESM.pdf]

Essential involvement of the CX3CL1-CX3CR1 axis in bleomycin-induced pulmonary fibrosis via regulation of fibrocyte and M2 macrophage migration

Yuko Ishida[1], Akihiko Kimura[1], Mizuho Nosaka[1], Yumi Kuninaka[1], Hiroaki Hemmi[2], Izumi Sasaki[2], Tsuneyasu Kaisho[2], Naofumi Mukaida[3], Toshikazu Kondo[1]*

[1]Department of Forensic Medicine, Wakayama Medical University, Wakayama, Japan

[2]Department of Immunology, Institute of Advanced Medicine, Wakayama Medical University,

[3]Division of Molecular Bioregulation, Cancer Research Institute, Kanazawa University, Kanazawa, Japan

Correspondence to: Toshikazu Kondo MD & PhD, Department of Forensic Medicine, Wakayama Medical University, 811-1 Kimiidera, Wakayama, 641-8509 Japan

Phone & Fax: +81-73-441-0641

E-mail: kondot@wakayama-med.ac.jp

Running title: The roles of CX3CR1 in bleomycin-induced pulmonary fibrosis

Key words: CX3CL1, CX3CR1, bleomycin, pulmonary fibrosis, fibrocytes

Supplemental table 1. Sequences of primers used for real-time RT-PCR

Transcript	Sequence
Cx3cl1	(F) 5’- ACCTATGGCCCTGACATCATCAC -3’
	(R) 5’- CTTGCCAGCCCTCAGAATCAC -3’
Cx3cr1	(F) 5’- CCGGTCTCATTTGCAGGCTTA -3’
	(R) 5’- CTTGCCAGCCCTCAGAATCAC -3’
Ccl2	(F) 5’-GCATCCACGTGTTGGCTCA-3’
	(R) 5’-CTCCAGCCTACTCATTGGGATCA-3’
Ccl3	(F) 5’-TGAAACCAGCAGCCTTTGCTC-3’
	(R) 5’-AGGCATTCAGTTCCAGGTCAGTG-3’
Ccl4	(F) 5’-CCATGAAGCTCTGCGTGTCTG-3’
	(R) 5’-GGCTTGGAGCAAAGACTGCTG-3’
Ccl5	(F) 5’-AFATCTCTGCAGCTGCCCTCA-3’
	(R) 5’-GGAGCACTTGCTGCTGGTGTAG-3’
Cxcl12	(F) 5’-GACTTCTACCCTCGCCAAGTTCC-3’
	(R) 5’-ACACTGAACCCATCGCTGCTTA-3’
Ccr1	(F) 5’- GGTTGGGACCTTGAACCTTG -3’
	(R) 5’- GGGTAGGCTTCTGTGAAATCTG -3’
Ccr2	(F) 5’- GAGGCTGTCAGGACTGAGTGAGA -3’
	(R) 5’- ATTTGAGAGCCCTGCTCACTTTC -3’
Ccr5	(F) 5’-GGTTCCTGAAAGCGGCTGTAAATA-3’
	(R) 5’-CTGTTGGCAGTCAGGCACATC-3’
Cxcr4	(F) 5’- GCCATGGCTGACTGGTACTTTG -3’
	(R) 5’- CAGGATGAGAACGCTGCTGTAGA -3’
Tgfb1	(F) 5’-GTGTGGAGCAACATGTGGAACTCTA-3’
	(R) 5’-TTGGTTCAGCCACTGCCGTA-3’
Col1a1	(F) 5’-ATGCCGCGACCTCAAGATG-3’
	(R) 5’-TGAGGCACAGACGGCTGAGTA-3’
Actb	(F) 5’-CATCCGTAAAGACCTCTATGCCAAC-3’
	(R) 5’-ATGGAGCCACCGATCCACA-3’

(F): Forward primer, (R): Reverse primer

Legends to Supplemental Figures

Supplemental Figure 1. (a) and (b), Double-color immunofluorescence analyses were performed as described in Materials and Methods. The immunopositive reactions to CX3CL1 were not detected on α-SMA$^+$ fibroblasts (a) and epithelial cells (b). Representative results from six individual animals are shown. Signals were merged digitally.

Supplemental Figure 2. Evaluation of leukocyte subsets in BALF of WT and *Cx3cr1*$^{-/-}$ mice. BALF was obtained from WT and *Cx3cr1*$^{-/-}$ mice at 5 days after BLM challenge. Single cell suspensions were stained with anti-CD3, anti-CD4, anti-CD8, anti-NK1.1, anti-Gr-1, and anti-CD11b, followed by flow cytometric analyses. Values represent mean±SEM (n=6).

Supplemental Figure 3. Intrapulmonary gene expression of chemokines and chemokine receptors in WT and *Cx3cr1*$^{-/-}$ after BLM challenge. The mRNA expression of each molecule was analyzed by quantitative RT-PCR as described in Materials and Methods. Values represent mean±SEM (n=6).

Supplemental Figure 4. Quantitative evaluation of fibrocytes in BM and peripheral blood by flow cytometric analysis before and at 12 days after BLM challenge. (a) and (c) Representative results on BM (a) and peripheral blood (c) from six independent experiments are shown here. (b) and (d) Changes in percentage of fibrocytes were determined in BM (b) and peripheral blood (d). Values represent mean±SEM (n=6).

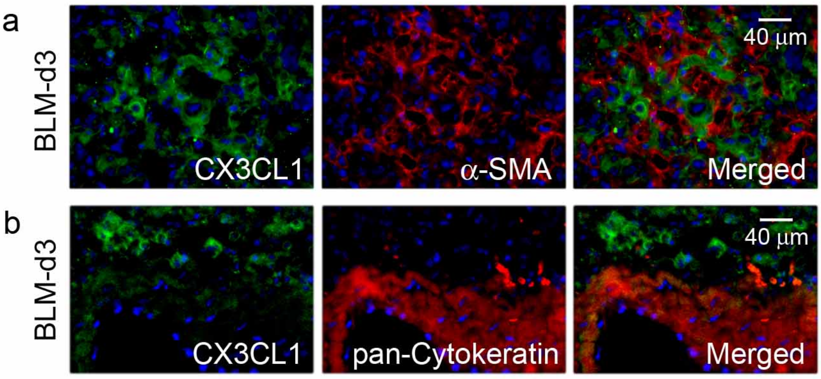

Supplemental Figure 1. (a) and (b), Double-color immunofluorescence analyses were performed as described in Materials and Methods. The immunopositive reactions to CX3CL1 were not detected on α-SMA^{+} fibroblasts (a) and epithelial cells (b). Representative results from six individual animals are shown. Signals were merged digitally.

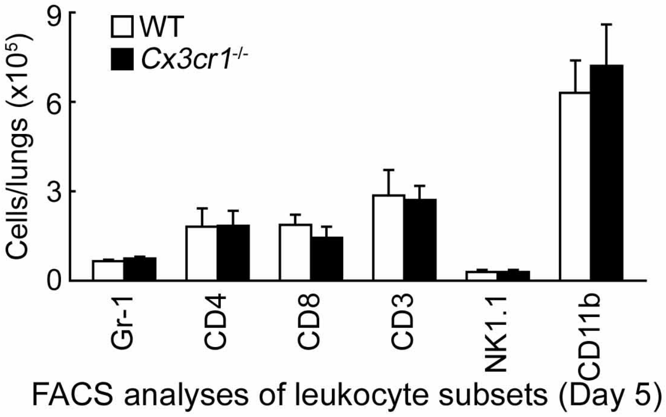

Supplemental Figure 2. Evaluation of leukocyte subsets in BALF of WT and *Cx3cr1*$^{-/-}$ mice. BALF was obtained from WT and *Cx3cr1*$^{-/-}$ mice at 5 days after BLM challenge. Single cell suspensions were stained with anti-CD3, anti-CD4, anti-CD8, anti-NK1.1, anti-Gr-1, and anti-CD11b, followed by flow cytometric analyses. Values represent mean±SEM (n=6).

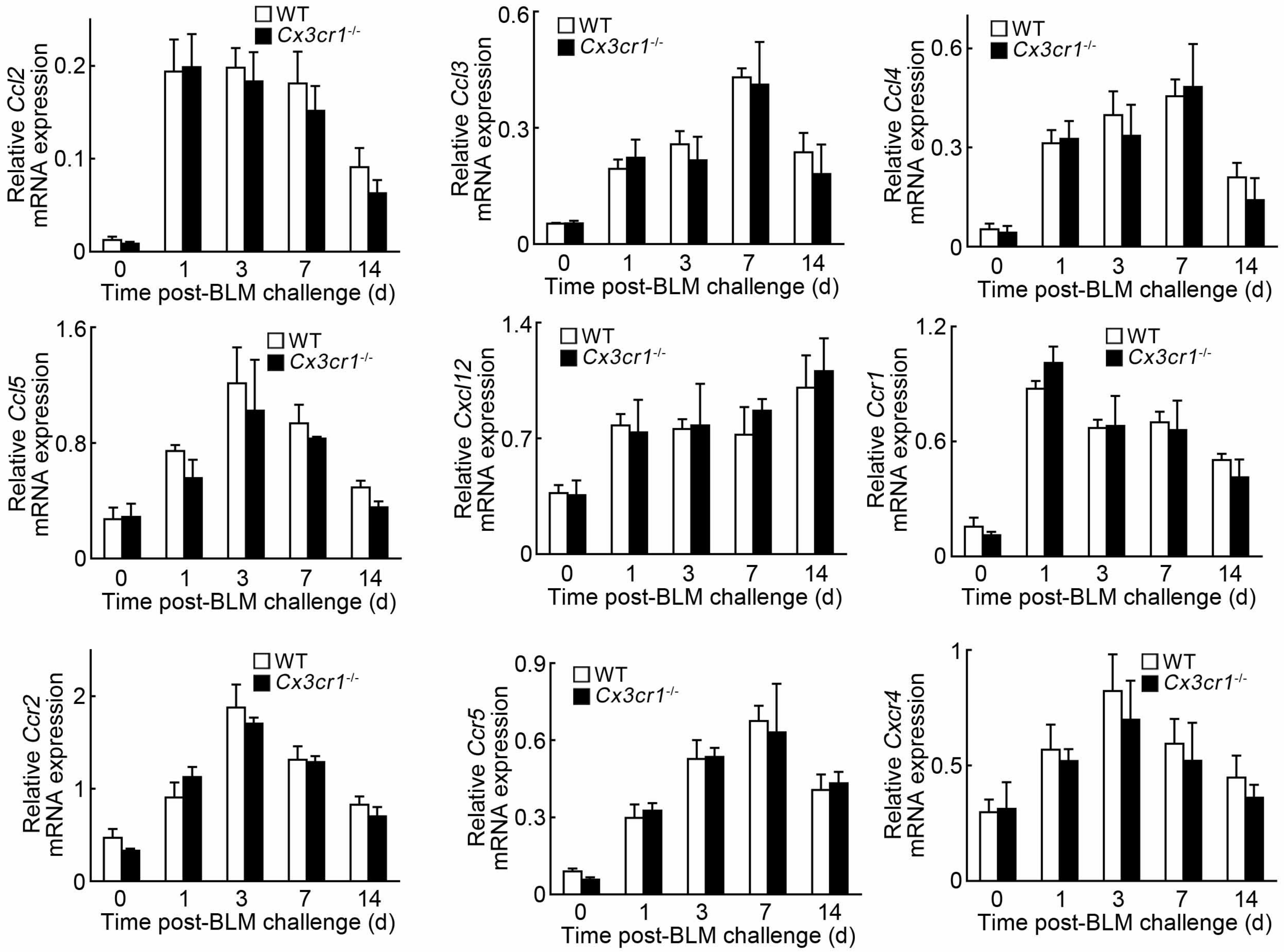

Supplemental Figure 3. Intrapulmonary gene expression of chemokines and chemokine receptors in WT and *Cx3cr1*$^{-/-}$ after BLM challenge. The mRNA expression of each molecule was analyzed by quantitative RT-PCR as described in Materials and Methods. Values represent mean±SEM (n=6).

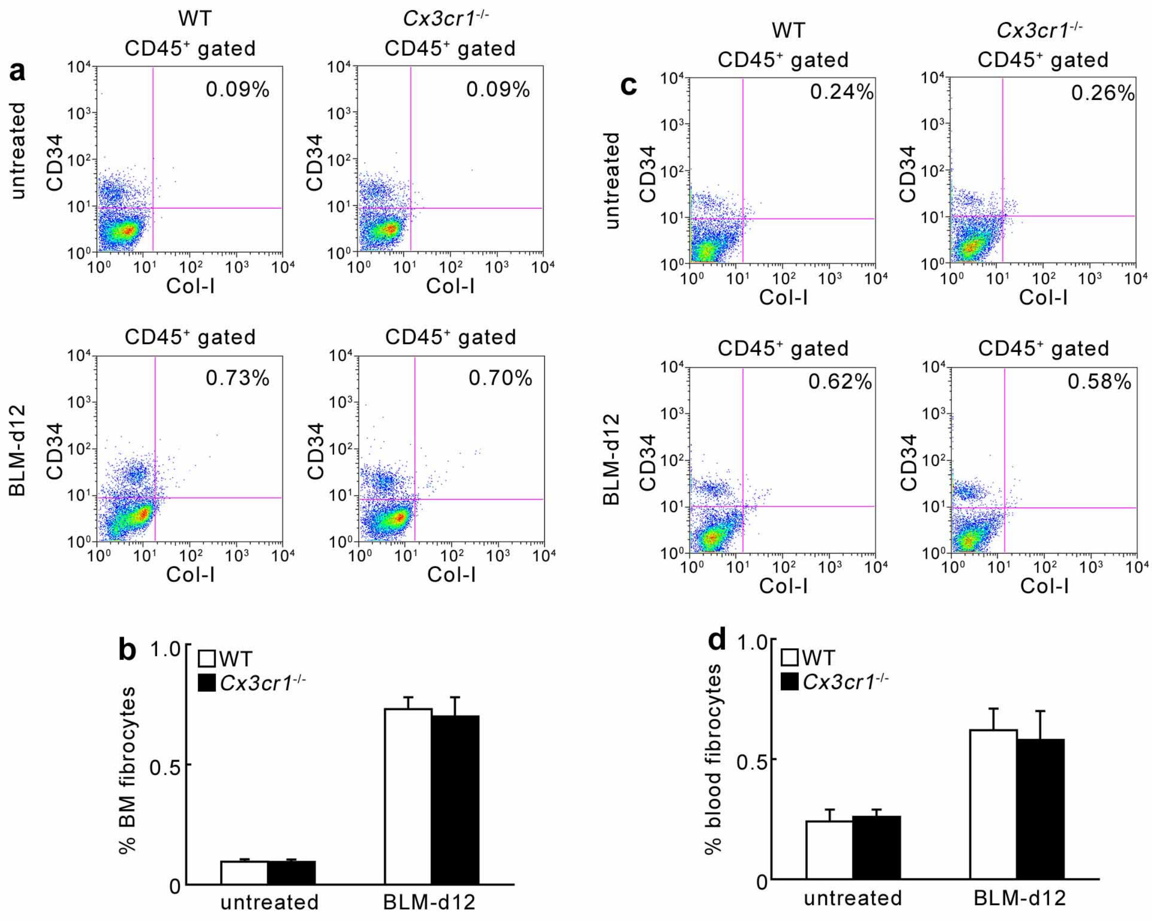

Supplemental Figure 4. Quantitative evaluation of fibrocytes in BM and peripheral blood by flow cytometric analysis before and at 12 days after BLM challenge. (a) and (c) Representative results on BM (a) and peripheral blood (c) from six independent experiments are shown here. (b) and (d) Changes in percentage of fibrocytes were determined in BM (b) and peripheral blood (d). Values represent mean±SEM (n=6).
